# Elevated carcinoembryonic antigen and bronchial obstruction caused by a rotten vegetable leaf mimic lung cancer: A case report

**DOI:** 10.1002/jcla.23579

**Published:** 2020-09-22

**Authors:** Ye Sun, Yan L. Ge, Li Q. Li, Yang Liu, Yang Lu, Qin Jing, Yi Chen, Wen Q. Li, Lin L. Hou, Pei Min

**Affiliations:** ^1^ Department of Respiratory Medicine North China University of Science and Technology Affiliated Hospital Tangshan China

**Keywords:** bronchoscopy, carcinoembryonic antigen, foreign body aspiration, lung cancer

## Abstract

**Background:**

Tracheobronchial foreign body aspiration is a potentially risky medical event, while the condition often requires early detection and rapid intervention to improve respiratory symptoms and prevent major morbidity. Notably, foreign bodies may not be identified and they are likely to be mistaken for neoplastic lesions. However, CEA, as one of tumor markers, presents to be available for assisting in lung cancer diagnosis, especially for non–small‐cell lung cancer, while the specificity of CEA is not high.

**Methods:**

Here, we described a case of bronchial opening obstruction with elevated carcinoembryonic antigen (CEA) that was firstly misdiagnosed as lung cancer and proved as foreign body aspiration in the upper lobe bronchus of right lung by bronchoscopy.

**Results:**

Carcinoembryonic antigen level increased. CT scan demonstrated a cavitation accompanied by multiple small nodular shadows appeared in the right upper lobe field. Bronchoscopy suggested right upper lobe bronchus was blocked by a brown smooth organism with plenty of purulent materials, which was proved as a rotten vegetable leaf.

**Conclusions:**

Elevated CEA and bronchial obstruction are not typical manifestations of lung cancer. Bronchoscopy is crucial for making a reliable diagnosis.

## 1 INTRODUCTION

Tracheobronchial foreign body aspiration is a potentially risky medical event, associating with clinical manifestations varying from acute respiratory distress, to chronic pulmonary infections, even death.^1^ Notably, accidental foreign body aspirations in children and adults with normal bronchial system are relatively uncommon occurrences,^2^ which can develop with or without the presence of obvious incentives. Therefore, the condition often requires early detection and rapid intervention to improve respiratory symptoms and prevent major morbidity.^1^, ^2^

Here, we present an adult case of bronchial opening obstruction in right upper lobe caused by a rotten vegetable leaf that was firstly misdiagnosed as lung cancer, which proved as foreign body aspiration in the upper lobe bronchus of right lung by bronchoscopy. The aim of this report was to provide clues that may facilitate a reliable diagnosis.

## 2 CASE REPORT

**Figure 1:**
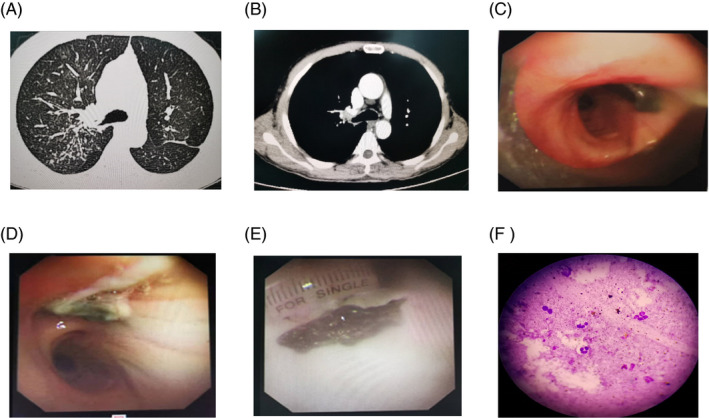
A‐B, Chest CT scan revealed a cavitation accompanied by multiple small nodular shadows appeared in the right upper lobe, the right upper lobe bronchus was in poor patency, and multiple mediastinal lymphadenopathies were seen. C‐D, Bronchoscopy suggested plenty of purulent materials in the bronchial wall of right upper lobe bronchus, of which the opening was completely blocked by the new organism. E, The new organism was discovered to be a rotten vegetable leaf. F, The new organism was clamped, and there were only scattered hyperchromatic cells with small nuclei but no tumor cells seen under rapid on‐site evaluation (ROSE).

In this article, we report an adult case with repeated pulmonary infection within a month firstly misdiagnosed as lung cancer, which confirmed as foreign body aspiration in the right upper lobe bronchus by bronchoscopy. In the absence of obvious incentives, the patient, a 57‐year‐old man, suffered from recurrent cough, yellow sputum, and occasional hemoptysis accompanied by fever for a month. He presented a history of smoking more than 40 years (about 20 cigarettes per day) with heavy alcohol intake. He received anti‐inflammatory treatment without significant relief. When he was transferred to our hospital for further treatment and admitted in our department, he received laboratory tests and chest CT scan. The physical examination was normal and there was no obvious abnormality in blood routine, while there was an obvious rise in CEA level (17.11 μg/L, normal value <5.0 μg/L). The chest CT scan revealed a cavitation accompanied by multiple small nodular shadows appeared in the right upper lobe field, the right upper lobe bronchus was in poor patency, and multiple mediastinal lymphadenopathies were pointed out during the observation period (Figure 1A‐B). Considering clinical manifestations combined elevated CEA and radiographic findings, lung cancer was highly suspected. Therefore, rigid bronchoscopy was arranged to identify the property of lesion. Bronchoscopy suggested plenty of purulent materials in the bronchial wall of right upper lobe bronchus, of which the opening was completely blocked (Figure 1C‐D). We used biopsy forceps to remove purulent secretion; then, we saw a brown smooth organism completely blocked the right upper lobe bronchus. We repeatedly clamped the new organism, but no tumor cells were seen under rapid on‐site evaluation (ROSE). So, we used carbon dioxide instead to freeze the foreign body. Eventually, the new organism was removed and the right upper lobe bronchus was unobstructed; then, we found the new organism was a rotten vegetable leaf (Figure 1E‐F). Hence, the definitive diagnosis was foreign body aspiration in the right upper lobe bronchus. We repeatedly asked the patient whether he ever had choking cough, while he had no history. After the operation, the patient pulmonary infection was under control and CEA level was normal after 2 weeks later (3.10 μg/L).

## 3 DISCUSSION

FB aspiration in adults, accounting for 25% of accidental aspiration cases, while the events mainly occurs in children. Symptoms of adult FB inhalation typically present with a sudden chocking and recurrent cough.^3^, ^4^ Due to non‐specific symptoms, initial occurrence usually goes unnoticed in elderly patient with altered state of awareness and misdiagnosed other chronic respiratory illnesses, such as COPD and asthma.^4^ When the diagnosis was delayed, it may cause severe harms to respiratory passageways, such as suffocation or death.^5^ Therefore, the condition often requires early detection and rapid intervention to improve respiratory symptoms and prevent major morbidity.

The pathogenesis of the events with aspects varies, including aspiration, ingestion, and purposeful insertion.^6^ Owing to the vertical orientation, a large majority of FBs become lodged in the right bronchial tree, including intermediate bronchi and the right lower lobe.^4^ Clinical heterogeneity of ingested foreign bodies can be associated with varying type, age, and geographic locales.^7^ In view of foreign body types, organic substances (such as peanut) can be aspirated most commonly in adults. Here, we present an adult case of bronchial opening obstruction in right upper lobe caused by a rotten vegetable leaf. Some other inorganic items (eg, plastic caps, nails, teeth) also can be aspirated, even if the event is an uncommon clinical entity in adults. For example, objects were mostly inorganic and represented by animal bones and cherry nuclei in Croatia. As for age, in most studies, the median age of those affected is between 50 and 60 years, and the risk of similar events is growing with aging. The elderly were less likely to recall the incident of aspiration; surprisingly, mostly 70% of the patients could not provide a clear medical history consistent with inhalation events before bronchoscopy, while there are no obvious risk factors in 10% of adult patients.^4^ In this article, the man had absence of obvious incentives. Geographically, in China, eating with chopsticks adds the risk of foreign body inhalation. In the Middle East, element of aspiration danger is attributed to headscarf needle stuck between the teeth.

Bronchoscopy designed initially as a safe and efficient tool to assist in removing foreign body.^8^ During bronchoscopy, a foreign body perhaps can be seen directly, or appears as tissue reaction characterized by granulation tissue, endobronchial stenosis, and edema. In 1897, Gustav Killian performed a bone extraction successfully using an esophagoscope and introduced bronchoscopy heralding a new era in removal of airway FBs.^4^ There are reports that the proportion of FB aspiration in adults has reached 0.16%‐0.33% percent in adult bronchoscopic procedures. Before 1970s, rigid bronchoscope was considered to be the procedure of choice. As technology advanced, with the aid of dedicated flexible instruments, flexible bronchoscopy could clear foreign bodies lodged more distally and expanded the bronchoscopic intervention scope in the surrounding airway. Increasingly, it boosted the success rate in identifying and removing the FBs and replaced rigid bronchoscopy as a highly applied technique in grown‐up, while rigid bronchoscopy is a preferred method when a foreign body was surrounded by granulation tissue and could not be gripped with flexible forceps.

Sometimes, the FBs could be detected on chest radiographs or CT scans, which usually provide a proper evaluation of the specific shape and location of foreign matter, ^9^ or reveal indirect signs, including non‐dissolving pneumonia, localized bronchiectasis, and atelectasis, especially airway obstruction.^7^ Occasionally, on the image studies, lung cancer is poorly differentiated from benign lung disease with nodular shadows.^9^ In this case, the patient had no clear history of aspiration; combined with elevated CEA and unsatisfactory therapeutic effects, we highly suspected the cavitation with nodular shadows as neoplastic lesions and linked it to lung cancer.^10^, ^11^ However, rigid bronchoscopy was arranged to discover the new organism to be a rotten vegetable leaf completely blocked the right upper lobe bronchus finally. Accordingly, foreign matter may not be identified and they are likely to be mistaken for neoplastic lesions in imaging.^12^, ^13^ As a result, identification of FB aspiration should be raised the profile and included in the differential diagnosis, especially for the elderly without a history of inhalation. Remarkably, positive CEA yields strong supports for lung cancer in patients with partially solid lesions. Increased level of CEA is probably traceable in many factors, among which several malignant diseases, particularly colorectal carcinoma, being discovered in 1965 by Gold and Freedman. But non‐malignant conditions such as hepatitis and bronchitis can also occur.^14^, ^15^ Additionally, Molina and other scholars' research on tumor markers indicated that the sensitivity is not high in lung cancer diagnosis with only increased CEA, especially for non–small‐cell lung cancer. It is concluded that CEA is not a unique marker for the lung cancer.^9^, ^16^, ^17^ In this case, after the operation of removing the foreign body, the patient pulmonary infection was under control and CEA level was normal after 2 weeks later. Bronchoscopy is the procedure of choice for confirming the diagnosis.

From this patient, our lesson is that initially we just regarded the cavitation with nodular shadows as a neoplastic lesion and did not be alert to the possibility of bronchial foreign body inhalation. Patients with high suspicion of malignant disease require bronchoscopy, as it is being applied today for abundant chest pathologies.^8^, ^13^

## 4 CONCLUSION

When a patient has repetitive respiratory symptoms combined abnormal changes on laboratory examinations and imaging, physicians should attach importance to if those changes are secondary to possible factors of tracheobronchial stenosis, especially guard against foreign body obstruction and tumor. Bronchoscopy is crucial for making a reliable diagnosis and treatment method.

## Supporting information

Supplementary Material
